# Out-of-pocket expenditure on maternity care for hospital births in Uttar Pradesh, India

**DOI:** 10.1186/s13561-018-0189-3

**Published:** 2018-02-27

**Authors:** Srinivas Goli, Anu Rammohan, Moradhvaj

**Affiliations:** 1 Room, 102 Centre for the Study of Regional Development (CSRD), School of Social Sciences (SSS), Jawaharlal Nehru University (JNU), New Delhi, India; 2 Discipline of Economics, University of Western Australia, M251, 35 Stirling Highway, Crawley, WA 6009 Australia

**Keywords:** Maternal health care, Catastrophic out-of-pocket expenditure, Health policy, UP, India

## Abstract

**Background and Objective:**

The studies measured Out-of-Pocket Expenditure (OOPE) for hospital births previously suffer from serious data limitations. To overcome such limitations, we designed a hospital-based study for measuring the levels and factors of OOPE on maternity care for hospital births by its detailed components.

**Methods:**

Data were collected from women for non-complicated deliveries 24-h before the survey and complicated deliveries 48-h prior to the survey at the hospital settings in Uttar Pradesh, India during 2014. The simple random sampling design was used in the selection of respondents. Bivariate analyses were used to estimate mean expenditure on Antenatal care services (ANCs), Delivery care and Total Maternity Expenditure (TME). Multivariate linear regression was employed to examine the factor associated with the absolute and relative share of expenditure in couple’s annual income on ANCs, delivery care, and TME.

**Results:**

The findings show that average expenditure on maternal health care is high ($155) in the study population. Findings suggest that factors such as income, place, and number of ANCs, type, and place of institutional delivery are significantly associated with both absolute and relative expenditure on maternity care. The likelihood of incidence of catastrophic expenditure on maternity care is significantly higher for women delivered in private hospitals (β = 2.427, *p* < 0.001) compared to the government hospital (β = 0). Also, it is higher among caesarean or forceps deliveries (β = 0.617, *p* < 0.01), deliveries conducted on doctor advise (β = 0.598, *p* < 0.01), than in normal deliveries (β = 0) and self or family planned deliveries (β = 0).

**Conclusions:**

The findings of this study suggest that the OOPE on maternity care for hospital births reported in this study is much higher as it was collected with a better methodology, although with smaller sample size. Therefore, ongoing maternity benefit scheme in India in general and Uttar Pradesh in particular need to consider the levels of OOPE on maternity care and demand-side and supply-side factors determining it for a more effective policy to reduce the catastrophic burden on households and help women to achieve better maternity health outcomes in poor regional settings like Uttar Pradesh in India.

## Background

According to World Health Organization (WHO)‘s Global Health Expenditure Database [1], Out-Of-Pocket Expenditure (OOPE) in India is among the highest in the world and even higher than many developing countries in Africa and Asia which have lower economy size and economic growth [2]. WHO reports that almost 86% of total healthcare expenditure in India involves OOPE incurred by households. The estimates from the Ministry of Health and Family Welfare (MoHFW), Government of India, also suggest that this figure could be around 71% [3]. The nature of expenditure on maternal health care is not an exception as a major share of it comes from OOPE of the households [4–7]. High maternity-related health care (Antenatal Care Services [ANCs], Delivery and Postnatal Care Services [PNCs]) expenditure is often considered as an important barrier in the utilization of health care during pregnancy and childbirth which may also be catastrophic for households [5, 8–11].

Studies from a wide range of settings have identified OOP expenditure as a risk factor of catastrophic financial burden on households (Roy and Howard [12]; Van Doorslaer et al. [13] for a range of Asian countries; Limwattananon, Tangcharoensathien and Prakongsai [14] for Thailand; Arsenault et al. [15] for Mali; Raban, Dandonaa, and Dandonaa [16]; Ghosh, 2010 [17] for India). Although, there is large literature from India that focuses on maternal health care, maternal mortality and its determinants [4, 8, 18–25], the empirical evidence on OOP healthcare expenditure during pregnancy and childbirth using primary data is relatively limited. A majority of the previous studies, unlike ours, were not conducted in hospital settings and were based on retrospective data that are known to be profoundly affected by recall bias and collected at the aggregated level [6, 9, 10, 26, 27].

In this study, we use the information on expenditure collected in the form of disaggregated components of maternity care from newly delivered mothers in a hospital setting and covers a wide range of demand and supply side factors determining the expenditure. Maternity expenditure includes not only institutional delivery expenditure but also covers expenditure on ANCs and PNCs. Thus, this paper aims to provide a comprehensive analysis of OOPE on maternity care for hospital births in the case of Uttar Pradesh (UP), one of the poorest socio-economic settings in India. This study has three specific aims: firstly, to provide estimates of maternity expenditure by its key components such as ANCs and institutional delivery in detail in terms of doctor fees, medication and transportation costs, and room rent; secondly, to assess the role of socio-economic and institutional factors in influencing the expenditure on maternal health care; third, to measure the extent of catastrophic expenditure on maternal health care by key background characteristics.

A reduction in global maternal mortality to below 70 per 100,000 live births by 2030 is a key target for measuring progress in Goal-3 of the United Nations’ newly adopted Sustainable Development Goals [28]. However, despite a decline of 45% in global Maternal Mortality Ratios (MMR) since 1990, still, there is a significant regional inequality. In 2013, Sub-Saharan Africa and South Asia accounted for 62 and 24% of all global maternal deaths, respectively, with India at 17% (50,000) and Nigeria at 14% (40,000) together accounted for one-third of all global maternal deaths [29].

Although MMR was estimated to be 159 per 100,000 live births in 2011–13 as the all India average, there is considerable heterogeneity across different states. While states such as Assam and UP have high MMR account for 300 and 285 maternal deaths per 100,000 live births, respectively; while it is only 61 deaths per 100,000 live births in Kerala [30]. In the absolute terms, UP is accounting for nearly a quarter of all maternal deaths in India [30].

In an outset, UP, the area of study was one of the states targeted for interventions under the Maternal and Child Health Sustainable Technical Assistance and Research (MCH-STAR) initiative to improve maternal, neonatal, child health, and nutrition policies and programmes in India [31–33]. The latest report of National Family Health Survey (NFHS) [34] shows that UP has around 67% deliveries have taken place in the institutions, and only 6% women have full ANCs. The lack of birth preparedness may be attributed to poor quality of maternal health care in government hospitals and relatively high costs in the private sector. Poor prenatal care and lack of birth preparedness have led to an increase in caesarean deliveries and the proportion of deliveries in private health centers [17, 31, 34, 35]. The previous studies attributed high maternal mortality and poor maternal health care to meager health expenditure by the state, highlighting the need for a more focused study of disadvantaged groups.

### Previous literature

The catastrophic levels and nature of maternity expenditure, especially about delivery expenditure have been documented in previous studies in India [6, 9, 10, 27, 34, 36], but their analyses suffer from data related limitations, especially in terms of completeness of details on maternity expenditure and also in terms of identifying the supply-side factors determining it. Most of these studies have used information only on expenditures incurred during delivery (from secondary datasets) to assess the OOPE on maternal health care, while in the case of supply-side factors determining it, they have focused on the role of specific government programmes such as the Janani Suraksha Yojana (JSY), a conditional cash transfer scheme to promote institutional deliveries in India. JSY is fully centrally sponsored programme which gives a mother package of Rs. 1400 ($23) cash assistance in rural areas and 1000 in urban areas in low performing states, while Rs. 700 ($10.98) and Rs. 600 ($10) for rural and urban areas respectively in high performing states [5, 9, 26].

A previous study on maternity expenditure by Bonu et al. [9] had used data from 60th Round (2004–05) of National Sample Survey (NSS). Neither previous rounds nor the latest (71st) Round of NSS has collected the expenditure information in disaggregated components (doctor's fee, medicine cost, transport cost and other related expenditure) for all three maternal health care components (ANCs, Delivery care, and PNCs). Although the latest round of NSS provided detailed information only for institutional delivery care, these questions were included under injury section of the expenditure module in the schedule, thus, may lead to misleading responses. The lack of component-wise data on expenditure related to pregnancy and childbirth has often led to poor planning; particularly, in policy decisions on the role and level of funding for maternity care under government programs. Furthermore, NSS does not provide information about a number of supply-side factors that influence maternity expenditure [37, 38].

The other major study by Mohanty and Srivastava [26] found that OOPE in public health centers has declined over time, with OOPE delivery expenditure in public hospitals averaging approximately US$39 compared to US$139 in private hospitals in 2004–07. These estimates were from the District Level Household Survey (DLHS), and were captured based on two simple questions: 1) How much did it cost you for transportation to the health facility for delivery, and 2) How much expenditure did you incur for delivery excluding transport cost [39]. Analyses based on these questions may lead to an underestimation of the full expenditure on maternity care because these questions ignored the spending incurred during ANCs, PNCs and other miscellaneous health care during pregnancy. Furthermore, household surveys rely on retrospective information such as expenditure on institutional delivery based on birth histories going back to five or 10 years before the survey which may lead to recall bias. Thus, studies that analysed the retrospective information could have suffered from potential recall bias [9, 10, 26, 34]. While studies based on small-scale primary surveys although considered the detailed components of maternity expenditure but mostly are descriptive, especially lacks robust statistical assessment [6, 11, 27]. Therefore, we conceptualised and designed a study to overcome the limitations of the previous studies and provide a detailed account of levels and factors associated with TME. The following section presents the design of the study.

## Methods

### Data

Our analyses are based on a dataset of 230 new mothers who were interviewed in a hospital setting in UP for the project ‘*Understanding Pregnancy Nutrition and Health Care among Women in UP: a Hospital-Based Survey*’ jointly conducted by Giri Institute of Development Studies (GIDS), Lucknow, India, and The University of Western Australia, Australia. The survey was conducted between November 2014 to December 2014. Out of total 384 recognised hospitals in the city of Lucknow, around 150 hospitals have inpatient maternity services. We have put inclusion criteria for the hospitals. The hospitals which intake at least minimum three new cases of inpatient delivery care per day have been considered for the survey. Finally, three largest inpatient delivery care hospitals each from a Government-owned, Government aided, and Private owned hospital were selected: (i) a Government hospital with no formal charges for the treatment, (ii) a Government-aided hospital, charging nominal fees, and (iii) a Private maternity hospital. The selection of hospitals ensures the variation in the socioeconomic profile of respondents, and also heterogeneity in the quality of healthcare and expenditure.

We used stratified random sampling design to select newly delivered women in the hospital setting from two specified strata: i) women with normal and uncomplicated deliveries, and ii) women with caesarean or other complicated deliveries. Further, we used simple random sampling to select the required number of participants from each stratum. For uncomplicated and normal deliveries, we interviewed women who gave birth 24-h prior to the survey; and for complicated deliveries including caesarean or forceps, we interviewed women who had given birth 48-h before the survey. The total number of eligible women admitted for delivery in month-long visits to selected hospitals were 945 (N). The sampled population for the survey was 250 (n). Out of the total randomly sampled population (*n* = 250), our final sample size consisted of 230 women after excluding those 18 women who were reluctant to participate or dropped out in between the interview and two women with “Zero” expenditure. Therefore, the participation rate among those who found eligible for the survey was 92%.

### Measures

The outcome variables were measured from the detailed information on maternity expenditure (component wise) in the dataset. It also provided the elaborative details on respondent’s demographic and socioeconomic characteristics, along with detailed information on supply-side factors (access to government programs such as JSY, the number of ANC visits, quality of ANCs, type of hospital for delivery, distance to ANCs and delivery hospital). A detailed description of the definition and coding of the variables are presented in Appendix 1.

### Statistical analyses

CSpro and STATA 13 software packages were used in data entry, data processing, and data analyses. We have presented the mean expenditures on ANC visits and delivery care using bivariate cross-tabulation. Multivariate linear regression models were estimated for both the absolute and relative share of income spent on ANCs and delivery care. We modeled two types of dependent variables with three models each: (1) absolute maternity expenditure in ANCs, delivery care and TME as the dependent variable and (2) relative TME measured regarding the share of maternity care expenditure in total income as a dependent variable. Further, we used multivariate ordered probit regression estimates to examine the levels and factors associated with Catastrophic Maternity Expenditure (CME) at different thresholds based on the share of maternity care expenditure in total annual income. This method is a highly preferable procedure for the estimation of catastrophic expenditure in health when one has access to household income information (see [13]).

**Fig. 1:**
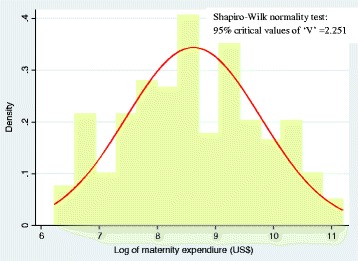
Shapiro-Wilk test of Normality and normal distribution curve for maternity care expenditure distribution

Since there are only two cases with “zero” OOPE in maternity care in the sample, so we preferred to use the general form of the linear regression model by dropping these two cases. The linear regression model is often adequate for analysis of health sector inequalities, where we simply want to predict discrete change in predicted probabilities of expenditure across the categorical variables, for example, maternity care expenditure conditional on income, age and so on. Given that, typically the distribution of maternity care expenditures is right-skewed, invariably the log of expenditure is modeled as a part of the linear regression model. The outcome variable was log-transformed and tested for normality using Shapiro-Wilk W test as suggested in the previous studies [40, 41]. The ‘V’ value of 2.251 suggests that the normality assumption for log-transformed total maternity expenditure can’t be rejected (see Fig. 1).

Given that the probability of the maternity expenditure (*y*_*i*_ > 0) is positive and determined by observable (*X*_1*i*_) and unobservable (ε_1*i*_) factors. This can be represented by an equation as below [13].

$$ E\left[\ln \left({y}_i\right)\left|{y}_i\right\rangle 0,{X}_{2i}{\beta}_2\right]=E\left[\ln \left({y}_i\right)\left|{X}_{1i}{\beta}_1+{\upvarepsilon}_{1i}\right\rangle 0,{X}_{2i}{\beta}_2\right]={X}_{2i}{\beta}_2 $$

Where ln (*y*_*i*_ > 0) is the log of positive maternity expenditure, *X*_2*i*_ is a vector of covariates; the term ε_2*i*_ includes unobservable factors, *E* is the expected level of medical expenditure.

## Results

**Fig. 2:**
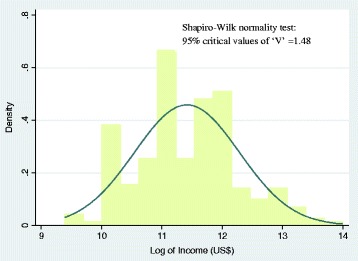
Shapiro-Wilk test of Normality and normal distribution curve for income distribution

Table 1 presents the descriptive statistics for key variables used in the analysis. Explanatory variables include the respondent’s socio-economic, demographic, public health and policy-related characteristics of women. The results show that the mean age of respondents is 26 years. About 46% of the respondents had up to higher education, 13% completed intermediate, with 40% having completed education at tertiary levels and above. Further, the findings show that the mean annual income of the couple is Rs. 2108 ($33). The normality test for income distribution was carried out through Shapiro-Wilk test. The results suggest that we cannot reject the null-hypothesis of non-normality with a value of ‘V’ at 1.498, which is also demonstrated through Fig. 2.

**Table 1:** Descriptive statistics of the study variables, *n* = 230

*Variable*	*Categories*	Mean/ Proportion	*Standard Error*
Age (in years)		26.04	0.2400
Education level of women	Up to high school	0.4696	0.0330
	Intermediate	0.1304	0.0223
	Under graduation and above	0.4000	0.0324
Religion	Hindu	0.8304	0.0248
	Muslim	0.1696	0.0248
Social group	SC/ST	0.1739	0.0250
	OBC	0.3652	0.0318
	General	0.4609	0.0329
Per capita annual income (Rs.)		2108.92	149.23
Place of residence	Urban	0.5913	0.0325
	Rural	0.4087	0.0325
Social networks	Yes	0.1000	0.0198
	No	0.9000	0.0198
Mass media exposure	No	0.1739	0.0250
	Yes	0.8261	0.0250
Number of previous pregnancies		1.0800	0.0600
Last pregnancy registered with ANM	Yes	0.3826	0.0321
	No	0.6174	0.0321
Number of ANC visits		5.15	0.2000
Quality of ANCs	Low	0.1609	0.0243
	Medium	0.1348	0.0226
	High	0.7043	0.0302
Place of ANCs	Government hospital	0.6565	0.0314
	Private hospital	0.3435	0.0314
Distance to ANC hospital		11.80	1.15
Who has taken decision on institutional delivery	Self/Family planned	0.5652	0.0328
	Doctor advised/ rushed to hospital due to EmOC	0.4348	0.0328
Type of delivery	Normal	0.4087	0.0325
	Caesarean/forceps	0.5913	0.0325
Type of hospital for delivery	Government hospital	0.4870	0.0330
	Government aided hospital	0.2652	0.0292
	Private hospital	0.2478	0.0285
Received JSY	Yes	0.313	0.0306
	No	0.687	0.0306
JSY amount ^a^		Rs. 1040.00	24.00

Note: ^a^ Estimate based on the sample who received the JSY by the date of survey

On an average, the number of previous pregnancies of women is one. The current pregnancy was registered with the ANC facility for 38% of the respondents. The average number of ANC visits among the respondents is five. They have traveled an average of 12 km to get their ANCs.

It is noteworthy that caesarean deliveries accounted for 55% of the institutional deliveries in the sample. The distribution of our sample across the different hospital types indicates that 49% went to purely government-owned hospital with no mandatory processing fee, which is relatively a bigger hospital, with the two other hospitals (Government aided and private hospitals) accounting for approximately 25% each. The government aided hospital was charging the nominal processing fee, while the private hospital was charging very high amounts compared to government hospitals. The delivery package in private hospital costs Rs. 14,000 ($220.4) to 60,000 ($944.4) depending on the type of complications women facing. Only 31% of women received the JSY benefit for institutional deliveries, with the average net benefits amount being Rs.1040 ($17). However, there is considerable variation observed in receiving net JSY benefits across the sample. It is varying between Rs.1400 ($23) to Rs.600 ($10). The average net benefit amount (Rs.1040 or $17) as found in this study is substantially below the government of India’s stipulated entitlement amount of Rs.1400 ($24) per institutional delivery.

Table 2 shows the average maternity expenditure by its components. The average expenditure on ANCs, institutional delivery and TME (i.e. include expenditures on both ANCs and delivery care) estimates are $56, $99 and $155, respectively. Even after adjusting for inflation using the Whole Sale Price Index (WPI) of 179.5 for the base year 2004–05 = 100 [42], the estimate for average total maternity expenditure is $86.

**Table 2:** Average maternity care expenditure by different components

*Variable*	*Mean in Rs.*	*Standard error*	*Mean in US$*	*Standard* *error*
*Outcome Variables (Maternity expenditure)*				
Doctor fee	246	83	4.01	1.34
Medicine	2068	230	33.65	3.74
Transport	465	77	7.57	1.26
Hospitalization charge/room rent	654	241	10.64	3.92
Total ANC expenditure	3433	398	55.87	6.47
Doctor fee	606	172	9.86	2.80
Medicine	2372	303	38.60	4.93
Transport	411	66	6.69	1.07
Hospitalization charge /room rent	1604	283	26.11	4.61
Other expenses	1104	142	17.96	2.31
Total expenditure of institutional delivery	6097	544	99.22	8.85
Total maternity expenditure (TME)	9530	811	155.09	13.20

### Absolute maternity expenditure

Table 3 presents the results of correlates of absolute maternity spending on ANCs, delivery care, and TME. The results show that social group, number of ANC visits and place of ANC visits emerge as significant correlates. In comparison with Scheduled Castes (SCs) / Scheduled Tribes (STs) (β = 0) and OBCs (β = 0.092), the absolute expenditure on ANCs is significantly higher among General Castes (β = 0.819, *p* < 0.01). The number of ANC visits (β = 0.113, *p* < 0.001) have shown a significant positive relationship with expenditure on ANC visits. Compared to government facility (β = 0), the absolute ANC expenditure is significantly higher in women who used private health facilities (β = 1.26, *p* < 0.001).

**Table 3:** Result of linear regression: Correlates of average maternity expenditure and share of maternity care expenditure in total couple’s annual income

*Variable*	*Categories*	Expenditure on maternity care			Percentage share of expenditure in couple’s annual income		
		*ANCs*	*Delivery*	*TME*	*ANCs*	*Delivery*	*TME*
Age (in years)		0.055(0.039)	0.053^**^(0.027)	0.028(0.028)	0.000(0.002)	0.001(0.002)	0.001(0.003)
Education level of women	Up to high school v/s Intermediate	−0.394(0.389)	−0.281(0.276)	−0.102(0.292)	0.002(0.018)	0.014(0.025)	0.017(0.035)
	Up to high school v/s Under graduation and above	−0.035(0.309)	−0.141(0.219)	−0.242(0.229)	0.055^***^(0.014)	0.069^***^(0.02)	0.120^***^(0.027)
Religion	Hindu v/s Muslim	0.249(0.351)	− 0.039(0.235)	0.184(0.253)	0.013(0.016)	0.016(0.021)	0.022(0.030)
Social group	SC/ST v/s OBC	0.092(0.379)	−0.201(0.259)	−0.111(0.27)	0.014(0.017)	0.023(0.023)	0.042(0.032)
	SC/ST v/s General	0.819^**^(0.38)	−0.131(0.266)	0.361(0.271)	0.022(0.017)	0.002(0.023)	0.021(0.033)
Per capita annual income		−0.207(0.172)	−0.324^***^(0.119)	− 0.237^*^(0.127)	−0.069^***^(0.008)	− 0.101^***^(0.011)	−0.177^***^(0.015)
Place of residence	Urban v/s Rural	0.085(0.271)	0.131(0.182)	0.154(0.197)	−0.025^**^(0.012)	−0.014(0.016)	− 0.042^**^(0.023)
Social networks	Yes v/s No	0.174(0.446)	−0.169(0.324)	0.229(0.326)	−0.027(0.02)	−0.005(0.028)	− 0.020(0.039)
Mass media exposure	No v/s Yes	0.139(0.401)	0.08(0.249)	0.251(0.264)	0.026(0.016)	−0.015(0.022)	0.017(0.031)
Number of previous pregnancies		−0.206(0.168)	−0.23^**^(0.115)	− 0.185(0.123)	0.010(0.008)	0.002(0.010)	0.013(0.015)
Last pregnancy registered with ANM	Yes v/s No	−0.382(0.256)	0.031(0.181)	−0.055(0.185)	−0.004(0.012)	0.015(0.016)	0.007(0.022)
Number of ANCs		0.113^***^(0.042)	–	0.070^**^(0.032)	0.000(0.002)	–	0.004(0.004)
Quality of ANCs	Low v/s Medium	0.315(0.487)	–	0.091(0.362)	−0.023(0.021)	–	−0.043(0.042)
	Low v/s High	0.443(0.39)	–	0.409(0.289)	0.009(0.016)	–	0.019(0.034)
Place of ANCs	Government hospital v/s Private hospital	1.26^***^(0.275)	–	0.331(0.252)	0.056^***^(0.013)	–	0.056^*^(0.030)
Distance to ANC hospital		0.000(0.004)	–	0.001(0.003)	0.000(0.000)	–	0.000(0.000)
Who has taken decision on institutional delivery	Self/Family planned v/s Doctor advised/ rushed to hospital due to EmOC	–	0.151(0.188)	−0.069(0.194)	–	0.011(0.016)	0.034(0.023)
Type of delivery	Normal v/s Caesarean/Forceps	–	0.244(0.19)	0.550^***^(0.195)	–	0.038^**^(0.016)	0.061^***^(0.023)
Type of hospital for delivery	Government hospital v/s Government aided hospital	–	0.597^**^(0.233)	0.814^***^(0.251)	–	0.035^*^(0.020)	0.051^*^(0.029)
	Government hospital v/s Private hospital	–	1.951^***^(0.250)	1.905^***^(0.307)	–	0.135^***^(0.022)	0.161^***^(0.036)
Received JSY	Yes v/s No	–	0.085(0.215)	−0.329(0.222)	–	−0.011(0.019)	−0.042(0.027)
Constant		6.256^***^(2.217)	9.885^***^(1.536)	8.457^***^(1.67)	0.781(0.101)	1.114^***^(0.137)	1.908^***^(0.201)
Prob > F		0.0000	0.0000	0.0000	0.0000	0.0000	0.0000
R-square		0.271	0.362	0.388	0.350	0.410	0.451
Adj R-square		0.202	0.304	0.320	0.296	0.362	0.451

Note: Significance levels: *p* < 0.05*, *p* < 0.01**, *p* < 0.001***, Standard error in parentheses

In case of absolute expenditure on delivery care, the age of women, the number of previous pregnancies, place of delivery emerge as significantly associated factors. The absolute maternity expenditure was increasing with increase in age of the women. Compared to Government hospital (β = 0), the absolute expenditure on delivery in Government aided hospital (β = 0.597, *p* < 0.01) and private hospital (β = 1.951, *p* < 0.001) is significantly higher.

**Fig. 3:**
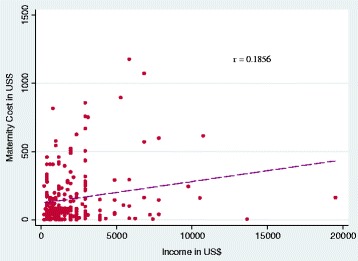
Relationship between maternity care expenditure and couple’s annual income

For absolute TME, income, the number of ANC visits, type of delivery and place of delivery are significant correlates. While income shows a negative relationship after adjusting all confounders. Moreover, bivariate analyses and correlation plot between income and TME show positive but not very strong relationship (Fig. 3 and Appendix 2). The number of ANC visits show a positive association with absolute TME. TME by the type of delivery indicates that caesarean or forceps delivery (β = 0.550, *p* < 0.001) incur higher expenditure compared to normal delivery (β = 0). By type of hospitals, in comparison with Government hospital (β = 0), the Government aided hopital (β = 0.814, *p* < 0.001) and private hospital (β = 1.905, *p* < 0.001) incur greater absolute maternity care expenditure. Except for education and income, the bivariate results reported in Appendix 2 are in line with the multivariate findings.

### Relative maternity expenditure

In case of relative TME (measured as maternity care expenditure share in couple’s annual income), the estimates from the multivariate linear regression model revealed that women’s education, place of residence, place of ANCs, type of delivery, type of hospital for delivery care remained significant predictors (Table 3). The probability of relative total expenditure on motherhood had significantly decreased with increase in income (β = − 0.177, *p* < 0.001). Compared to urban areas, the share of TME in income is slightly lesser in rural areas (β = − 0.042, *p* < 0.01). The relative TME by place of ANCs suggest that it is higher in private health facility (β = 0.056, *p* < 0.05) than in Government hospital (β = 0). With reference to normal deliveries (β = 0), the relative spending on caesarean or forceps deliveries were significantly higher (β = 0.061, *p* < 0.001). Similarly, with reference to deliveries in Governmnet hospital (β = 0), the relative TME was considerably high in Government aided hospital (β = 0.051, *p* < 0.05) and private hospitals (β =0.161, *p* < 0.001). Separate regression models for ANCs and delivery care expenditure reveal that education, income, place of residence and place of ANCs emerged as significant predictors of variation in relative expenditure on ANCs, while education, income, type of delivery and type of hospital for delivery emerged as significant predictors of variation in relative expenditure on delivery.

### Incidence of ‘catastrophic expenditure’

In Appendix 3, we present the estimates of the proportion of households with CME for hospital births at different thresholds by using both bivariate cross-tabulation. Our results show that around 26.5% of the households had incurred catastrophic expenditure at the threshold level of 15% and above in terms of share of TME in family annual income. At the threshold level of above 25% in share of maternity expenditure in the family annual income, about 13.5% of the families have incurred the catastrophic expenditure. Our analysis further shows that at above 25% threshold level, the catastrophic maternity expenditure by place of ANCs was considerably higher for women who have used private health facility (20%) compared to the governmental hospital (10.6%). The doctor’s advise for institutional delivery (16%) have resulted in higher catastrophic maternity expenditure than self or family planned institutional delivery (11.5%). Caesarean or forceps delivery have contributed 5% more catastrophic maternity expenditure than normal delivery. The catastrophic maternity expenditure in private (26.3%) and government aided (14.7%) was higher than government (6.2%) hospital.

The results of the order probit regression showing the correlates of the incidence of catastrophic maternity care expenditure at different threshold levels (0–15%, 15–25% and above 25%) are presented in Table 4. After controlling a number of confounding factors, the results reveal that with the increase of the income, household significantly less likely (β = − 1.435, *p* < 0.001) to face occurrence of catastrophic maternity care expenditure. While with the number of ANCs visits, the households more likely (β = 0.076, *p* < 0.01) to face incidence of catastrophic maternity care expenditure. The decision on institutional delivery by the doctor (β = 0.598, *p* < 0.01) associated with a greater incidence of catastrophic maternity care expenditure for women compared to self or family taking the decision (β = 0). The likelihood of catastrophic maternity expenditure is higher among caesarean or forceps deliveries (β = 0.617, *p* < 0.01) compared to normal deliveries (β = 0). By type of hospital for delivery, the results suggest the occurrence of catastrophic maternity care expenditure is higher in government aided (β = 0.571, *p* < 0.05) and private hospitals (β = 2.47, *p* < 0.001) than government hospital (β = 0).

**Table 4:** Results of Order probit regression: Correlates of catastrophic maternity expenditure at different threshold levels

*Variable*	*Categories*		Marginal effect		
		Coefficient	Less than 15% level	15–25% level	More than 25% level
Age (in years)		0.008(0.034)	−0.002(0.008)	0.001(0.006)	0.001(0.002)
Education level of women	Up to high school v/s Intermediate	−0.356(0.367)	0.076(0.067)	−0.055(0.051)	−0.021(0.018)
	Up to high school v/s Under graduation and above	0.319(0.286)	−0.080(0.076)	0.055(0.051)	0.025(0.026)
Religion	Hindu v/s Muslim	0.044(0.306)	−0.011(0.075)	0.007(0.052)	0.003(0.023)
Social group	SC/ST v/s OBC	0.246(0.334)	−0.062(0.087)	0.042(0.059)	0.019(0.029)
	SC/ST v/s General	0.162(0.333)	−0.040(0.082)	0.028(0.057)	0.012(0.026)
Per capita annual income		−1.435^***^(0.195)	0.350^***^(0.052)	−0.244^***^(0.049)	−0.106^***^(0.030)
Place of residence	Urban v/s Rural	0.279(0.242)	−0.066(0.056)	0.046(0.040)	0.020(0.017)
Social networks	Yes v/s No	0.163(0.45)	−0.040(0.109)	0.028(0.076)	0.012(0.033)
Mass media exposure	No v/s Yes	0.252(0.31)	−0.056(0.063)	0.04(0.046)	0.016(0.017)
Number of previous pregnancies		−0.038(0.154)	0.009(0.037)	−0.007(0.026)	−0.003(0.011)
Last pregnancy registered with ANM	Yes v/s No	0.102(0.227)	−0.025(0.055)	0.017(0.039)	0.008(0.017)
Number of ANCs		0.076^**^(0.037)	−0.018^**^(0.009)	0.013^*^(0.007)	0.006^*^(0.003)
Quality of ANCs	Low v/s Medium	−0.894*(0.457)	0.153^***^(0.054)	−0.115^***^(0.043)	−0.039^**^(0.016)
	High	−0.542(0.353)	0.147(0.104)	−0.096(0.066)	−0.050(0.042)
Place of ANCs	Government hospital v/s Private hospital	−0.156(0.302)	0.038(0.074)	−0.026(0.051)	−0.012(0.022)
Distance to ANC hospital		0.003(0.004)	−0.001(0.001)	0.000(0.001)	0.000(0.000)
Who has taken decision on institutional delivery	Self/Family planned v/s Doctor advised/ Rushed to hospital due to EmOC	0.598^**^(0.242)	−0.146^**^(0.059)	0.101^**^(0.044)	0.044^**^(0.021)
Type of delivery	Normal v/s Caesarean/forceps	0.617^**^(0.251)	−0.151^**^(0.059)	0.105^**^(0.044)	0.046^**^(0.021)
Type of hospital for delivery	Government hospital v/s Government aided hospital	0.571*(0.321)	−0.157(0.096)	0.102^*^(0.060)	0.055(0.040)
	Government hospital-I v/s Private hospital	2.427^***^(0.422)	−0.742^***^(0.095)	0.253^***^(0.052)	0.489^***^(0.116)
Received JSY	Yes v/s No	0.627(0.289)	0.153(0.070)	0.106(0.051)	0.046(0.024)
	Content 1	−12.798(2.488)			
	Content 1	−11.955(2.462)			
	Prob>chi2	0.0000			
	Pseudo R2	0.3436			
	Log likelihood	113.15			

Note: Significance levels: *p* < 0.05*, *p* < 0.01**, *p* < 0.001***, Standard error in parentheses

## Discussion

Using the hospital-based data, this study had comprehensively analysed the maternity healthcare expenditure for hospital births in an urban setting in India. Our analyses show that the average absolute maternity care expenditure of $154 is considerably high for women residing in a poor state like UP. Even after adjusting for inflation, our estimate of average delivery care expenditure ($56) is double in comparison with an estimate from the study by Mohanty and Srivastava [36] for UP, based on DLHS data ($23), while it is in line with recent NFHS-IV [32]. Also, our estimates of average delivery care expenditure are slightly more than estimates from the other study that used NSS data [9]. Furthermore, a comparison of average institutional delivery expenditure in the present study (both before [US$ 99.1] and after [US$56] inflation adjustment) to that of the previous studies from other countries suggests that it is much higher compared to delivery expenditure in developing countries like Kenya (US$18.4), Burkina Faso (US$7.9) and Tanzania (US$5.1), but much lower compared to estimates from developed countries such as Canada (US$ 2733) [43]. However, the sample of only 230 women from three select hospitals, although not insufficient but a small sample to do a more robust statistical estimates, is one of the major limitations of our study. Moreover, given the non-clinical nature of the survey, the study failed to account for the medical complications of the women during pregnancy and childbirth in detail. The methodology of the study can be replicated in the future with larger samples by giving importance to some of the non-clinically measurable medical conditions of the women in a greater detail. While the strength of this study is some the key supply-side correlates used to predict TME, were not used previousely in Inida.

Nevertheless, our findings are in tune with international literature which finds that CME occurs regardless of the amount of money paid for healthcare services because the capacity for health expenditure in low-income families is much lower compared to rich families [44]. For instance, we found that although absolute expenditure in higher income families is much higher compared to low-income families, in the case of CME, it is much higher in lower income families compared to higher income families. In UP, we also found that although maternity care expenditure has increased for both poor and rich families, the reasons behind it are different for the two groups. For poor women, factors such as poor antenatal care, lack of birth preparedness and emergency obstetric complications are important reasons for higher OOPE. While, in case of women from higher income families, they may be ready to pay more expenditure to receive better quality health care. Nevertheless, the supply-side factors such as the type of hospital for ANCs, the type of delivery and the type of hospital for delivery emerged as significant factors in terms of influencing the maternity expenditure among both poor and rich women.

However, the association between JSY incentives and maternity expenditure was insignificant after adjusting for other related predictors. This finding is important in the context that the JSY scheme was specifically launched to reduce the economic burden of out-of-pocket expenditures on health care during childbirth. Ineffectiveness of JSY as an instrument to reduce catastrophic expenditure for maternity care may be attributed to two reasons: (1) The leakages in the distribution of JSY entitlements and differential bargaining power of deprived and affluent social status families. Therefore, it reveals from our findings that the net JSY benefits received by women are very less compared to the amount prescribed in JSY entitlements; (2) Even if women accessing the full JSY entitlement (US$ 24), it is too less in comparison with average maternity or delivery expenditure (US$ 155). Thus, this study indicates that even the full amount under JSY entitlement is too meager to make any significant impact on the economic burden of maternity expenditure on the families. Some of the previous studies also raised their concern on the inadequate supply of quality service delivery in public health institutions, high costs of health care services in private institutions and ineffective demand-side financing in absence of quality health care system [5, 11, 25, 45–47].

Our findings assume a huge relevance in the context of recent WHO report on the increased rate of caesarean births and cost of institutional births in India [33]. The report indicated that the share of caesarean births cost is five times more than the normal delivery—and its rate has been doubled in past one decade. A report based on latest NFHS round four [34] suggests that caesarean delivery in urban private hospitals are as high as 45% in India and 37% in Uttar Pradesh, which is considerably higher than the recommended level of 10–15% by WHO [2]. In this context, findings from this study have huge implication for policy.

Our study suggests some key policy implications. First, it identified the components of maternity care expenditure which can help in the better planning of government services—answers question on where and on what we need to spend more. Second, poor access to public health services in rural areas or nearby towns may mean that women have to travel to the capital city in case of complicated deliveries, which as a result leads to high health care expenditure. Moreover, in anticipation of better quality services, women choose private hospitals for ANC and delivery care, which in turn contributes to higher OOPE. As the study found that there exist large disparities in the maternity care expenditure in public and private health facilities in UP, there is also a need to reduce the expenditure gap between these two types of services and also to increase the availability and accessibility of quality public health facilities for maternity care. Third, there is a need to increase transparency and reduce the complexities in the distribution of JSY entitlements for women. Fourth, the high proportion of caesarean deliveries as observed among the sample raises serious concerns of how ANCs can be improved to avoid unnecessary complications at the time of delivery and the catastrophic expenditure for maternity care.

## Conclusion

Findings from both previous studies [9, 36, 47] and the current analysis suggest that high OOPE on maternity care can be a serious constraint in utilizing the maternity care in developing countries. In this study, we had estimated only direct costs, but apart from women, some of their family members who accompanied her might have lost their wages during pregnancy and delivery care which is an add-on to household economic burden, especially among lower income groups. Moreover, due to hospital-based survey setting, we couldn’t account for postnatal expenditure through a follow-up survey which would have further added to the burden of maternity care expenditure in households. To avoid the burden of catastrophic expenditure on the families, India, and its states need to scale-up their maternity entitlements at par with OOPE on maternity care as evident in this study. The improvements in the quality of Maternity Benefit Package (MBP) services and the increase of JSY incentives not only to support delivery care expenditure but also to cover expenditure incurred on ANC, Internatal Care (INC), Postnatal Care (PNC), and Essential New Born Care (ENBC). The success of MBP services and its holistic benefits can only be achieved by the substantial increase in entitlements and comprehensive improvements in supply-side factors, especially budget sanction to maternity benefit scheme. Given the fact that both state and central public health expenditure as a percent of GDP in India (4%) is lowest not only among the larger economies but also significantly less compared to some of the poorest economies in the world such as Niger (6.6%), Sudan (6.6%), Malawi (8.4%) and Uganda (9.8%) [48–51]. Therefore, there is a need to prioritise public health expenditure to address the exceedingly high OOPE incurred by a large proportion of women for maternity care. Although, the government of India under National Food Security Act (NFSA) 2013 and Maternity Benefit Act 2016 has risen cash incentives to Rs. 6000, but it is not 100% centrally sponsored scheme. Therefore, many states are yet to implement it in its full spirit [47]. The new act clearly indicates payment of incentives in three installments first installment (Rs. 3000) at the first trimester of pregnancy, second (Rs. 1500) at the time of institutional delivery and third after 3 months of delivery (Rs. 1500). However, the evidence from the states which are implementing these schemes reveals that the payments are paid only after delivery which doesn’t help to improve the pregnancy outcomes because by the time women receive the money the damage for pregnancy already takes place [47]. Therefore, states should implement the scheme in its full spirit to achieve the holistic outcome of such policy.

## Appendix 1

**Table 5:** Description of study variable: Definition/Coding

Name of the variable	Definition/Coding
*Outcome variables*	
Maternity expenditures	Maternity expenditures were measured as a linear variable using three broad categories: ANC expenditures, delivery expenditures, and total maternity expenditures. Each of these broad categories was derived from the five disaggregated expenditure components: doctor’s fees, medication costs, transportation costs, the cost of hospitalization and room rent.
Catastrophic maternity expenditure [CME]	CME was measured in relation to income. However, there is no accepted single measure of catastrophic spending in the health financing literature. Some studies measure catastrophic spending in relation to the budget share (Wagstaff and van Doorslaer 1993; Russell 2004; Pradhan and Prescott 2002); while others argue that catastrophic spending should be measured in relation to capacity to pay, such as household expenditure net of food spending (Xu et al. 2003; Garg and Karan 2009; Raban et al. 2013). Nonetheless, all measures suggest that when households expand a large proportion of their budget on health care, they often forgo other goods and services, which can have the negative implications for their living standards (Raban et al. 2013). We use the most popular approach, which defines the medical spending as “catastrophic” if it exceeds some fraction of a household’s income or total expenditure in a given period, typically 1 year (Berkiw 1986; Wagstaff and van Doorslaer 1993; Wagstaff and van Doorslaer 2003; Russell 2004). We use income rather than consumption expenditure in the denominator. Since, there is no acceptable definition for *cut-offs*, we calculated catastrophic spending for different *cut-off* points (i.e. less than 15% level, 15 to 25% level and above 25%). This assessment of catastrophic spending at multiple *cut-offs* provides us with both the incidence as well as the intensity of catastrophic spending (O’Donnell et al. 2005).
*Predictor variables: Socio-economic*	
Age (in years)	Age of the women is categorised into three groups: less than 25 years, 25–29 years and above 30 years. This classification was done by keeping in the mind both the distribution of the sample across the ages and also considering the ideal ages of childbearing for better pregnancy outcomes. While for regression analyses we used age as linear variable.
Education level of women	The educational status of women is coded into three categories: up to high school, intermediate and under graduation and above. These groups are classified in such as way that they have a distinct effect on the nature of health care spending.
Religion	The presence of other religions in Uttar Pradesh is nearly negligible which is also reflected in our sample. Therefore, we have classified our sample into Hindu and Muslim.
Social group	The social groups are recoded into three groups: Scheduled Caste (SC)/Schedule Tribe (ST), Other Backward Castes (OBCs) and General Castes. A system that allows social hierarchal division of people in India.
Family’s annual per capita income	The collection of income of the household is always a challenging exercise. In the case of this survey, it is, even more, difficult because it was at the hospital setting. However, the 82% of our sample are coming from Urban and Semi-urban areas and more than 70% of the sample is from the non-primary sector as a principal occupation. Within primary sector (30%), 18% of them are daily wage labourers. Therefore, in total 88% (around 202 out 230) of our respondents have not faced any problem in reporting their daily or monthly or annual income. However, for those who stated their husbands/her own occupation as cultivation and business, we have asked women to take the help of family members (who were present with her at the time of survey, mostly the husband) in reporting the annual share of couples’ income in the total income of the household in past 12 months if in case they are residing in joint families. Thus, we have collected daily (a reference to last working day) or the monthly (reference to the past 1 month)/Yearly (a reference to the past 1 year) income of women and her husband but later it is aggregated to estimate the annual per capita income of the family members. Based on the distribution of family’s annual per capita income, we have categorised the income into four groups: Below Rupees (Rs.) 24,000 (Below $390), Rs. 24gmht000 to 60,000 ($390 to $976), Rs. 60,001 to 100,000 ($976.1 to $1626), Rs. Above 100,000 ($1626). The first category is near to below poverty line according to the World Bank definition of poverty line prior to 2015 i.e. less than 1.00$ per day. While for regression analyses we used income as a linear variable.
Place of residence	Place of residence is recoded into Urban and Rural area.
Social network	We collected information on social networks of the family with any medical person working in the hospital they have visited. The answer was recorded as “Yes” if they have social connection otherwise coded as “No”.
Any mass media exposure	Mass media exposure is a composite variable. It is computed based on women’s exposure to print media (newspaper/magazine), and electric media (television, radio, and cinema). Exposure to any of these media sources was denoted “Yes” Otherwise “No”.
*Predictors (Demographic/Public health/Policy)*	
Number of previous pregnancies	The number of previous pregnancies is a continuous variable that was recoded into 0 “for the first time pregnant” 1 “if the current pregnancy is the second” 2 “for more than two time pregnant women”. While for regression analyses we used it as linear variable.
Current pregnancy registered with the ANM	If the women were registered, their current pregnancy with ANM was coded as “Yes” otherwise “No”.
Number of ANC visits	The number of ANC visits is a continuous variable that was recoded into less than 3, 3 to 8, and 9 and above ANCs. Minimum three ANC visits are a part of World Health Organization standards of Full ANC. In case of regression analyses we used it as linear variable.
Quality of ANCs	The quality of the ANC is a composite indicator computed from the information on medical checkups conducted during ANCs. ANC cost is very sensitive to the kind of medical tests conducted during ANC visits that are in turn indicating the quality of medical check-up during ANC visits. We consider six check-ups and advice: weighing, blood pressure, blood test, urine test, abdomen check-up and ultrasound test, advice on food and personal care. Out of seven, no or less than 3 checkups is considered as “low quality” ANC, 3 to 4 checkups is considered as medium quality ANC, 4 and above checkups is considered as a high-quality ANC check-up.
Place of ANC	Place of the ANC is coded as Government health center/ Hospital and Private clinic/ hospital.
Distance to ANC clinic/ hospital	Distance to ANC hospital has a huge role in expenditure on transportation. We coded distance to ANC clinic/hospital into less than 3 km (km), 3.1 to 5 km, 6 km and above. For regression analyses we used it as linear variables.
Decision on institutional delivery	The decision on institutional delivery is coded as self/family planned, the doctor advised, rushed to the hospital due to Emergency Obstetric Care (EmOC). The rationale behind this classification is that if the delivery is pre-planned by a woman or her family often incurs less expenditure than doctor advised or rushed to a hospital in EmOC.
Type of delivery	Type of delivery has a huge impact on delivery cost. It is recoded as normal and caesarean or forceps.
Type of hospital for delivery	Type of hospital for delivery is coded into (I) Government Hospital, which is purely government, (II) a Government-aided hospital, which is government hospital, but charges a nominal fee, and (iii) a Private maternity hospital.
Received JSY	If women received JSY, it is coded as “Yes” Otherwise “No”.

## Appendix 2

**Table 6:** Mean expenditure (US$) and percentage share of maternal health care spending in couple’s annual income

*Variable*	*Categories*	Mean expenditure (US$)			Mean percentage share into couple annual income		
		*ANCs*	*Delivery*	*TME*	*ANCs*	*Delivery*	*TME*
Age (in years)	Less than 25	38.83(7.01)	86.51(11.73)	125.34(14.97)	3.29(0.57)	8.44(1.34)	11.72(1.62)
	25–29	47.82(6.95)	91.2(13.84)	139.02(18.24)	5.14(1.25)	7.99(1.45)	13.14(2.37)
	30 and above	105.33(24.91)	140.52(23.71)	245.87(43.49)	5.19(1.26)	9.95(2.53)	15.14(3.51)
Education level of women	Up to high school	30.1(5.3)	73.24(9.57)	103.35(12.18)	3.03(0.65)	7.3(1.06)	10.33(1.38)
	Intermediate	51.78(15.79)	68.66(14.5)	120.45(23.97)	3.93(1.03)	7.01(2.15)	10.94(2.88)
	Under graduation and above	87.35(13.37)	139.52(17.72)	226.88(27.11)	6.36(1.35)	10.48(1.86)	16.84(2.87)
Religion	Hindu	53.9(7.29)	99.85(10.12)	153.77(15.13)	4.34(0.72)	8.44(1.02)	12.77(1.49)
	Muslim	65.24(13.5)	95.72(16.5)	160.98(24.09)	5.2(1.43)	9.03(2.4)	14.22(3.62)
Social group	SC/ST	25.48(8.32)	80.41(16.96)	105.89(20.5)	3.08(1.45)	8.83(1.84)	11.9(2.62)
	OBC	40.75(7.17)	91.35(13.98)	132.11(18.69)	4.87(1.32)	10.14(1.89)	15.01(2.74)
	General	79.23(12.06)	112.41(14.29)	191.65(22.81)	4.7(0.75)	7.15(1.2)	11.85(1.82)
Per capita annual income	Below $390	54.26(13.76)	86.27(20.94)	140.53(28.66)	16.83(5.03)	24.64(5.61)	41.47(8.63)
	$390.1 to $976	39.08(8.73)	92.04(16.5)	131.13(22.96)	5.95(1.35)	13.83(2.54)	19.78(3.52)
	$976.1 and above	61.58(8.81)	103.23(11.44)	164.82(17.35)	2.34(0.29)	4.63(0.53)	6.97(0.68)
Place of residence	Urban	71.9(10.01)	107.72(12.99)	179.63(19.99)	5.4(0.99)	8.34(1.3)	13.74(2.01)
	Rural	32.58(5.6)	86.75(10.68)	119.34(13.66)	3.15(0.62)	8.82(1.33)	11.97(1.72)
Social networks	Yes	63.16(19.95)	124.76(34.17)	187.93(52.55)	3.46(1.81)	5.51(2.15)	8.97(3.95)
	No	55.01(6.84)	96.31(9.08)	151.33(13.47)	4.59(0.68)	8.87(1.02)	13.47(1.47)
Mass media exposure	No	16.68(4.14)	77.68(14.51)	94.36(16.82)	2.82(0.81)	10.6(2.75)	13.42(3.24)
	Yes	64.07(7.65)	103.67(10.25)	167.75(15.43)	4.83(0.76)	8.1(0.98)	12.93(1.53)
Number of previous pregnancies	0	73.08(12.94)	128.7(18.58)	201.79(26.52)	5.28(0.99)	9.83(1.83)	15.11(2.6)
	1	55.26(11.71)	96.86(16.47)	152.13(25.34)	3.63(0.83)	7.73(1.6)	11.36(2.16)
	2 and above	42.4(9.2)	77.12(11.24)	119.52(17.26)	4.46(1.28)	8.08(1.48)	12.54(2.33)
Last pregnancy registered with ANM	Yes	58.07(9.48)	95.68(14.74)	153.76(21.28)	5.35(1.08)	8.53(1.51)	13.87(2.41)
	No	54.44(8.69)	101.31(11.08)	155.75(16.87)	3.95(0.79)	8.54(1.2)	12.49(1.67)
Number of ANCs	Less than 3	39.15(15.8)	–	119.49(25.06)	2.27(0.65)	–	10.08(1.84)
	3 to 8	57.26(7.92)	–	157.71(16.39)	5.25(0.91)	–	14.3(1.9)
	9 and above	71.3(15.3)	–	189.51(39.22)	3.74(0.86)	–	10.76(2.81)
Quality of ANCs	Low	25.12(7.2)		110.03(23.35)	2.65(0.94)	–	9.71(2.18)
	Medium	23.77(6.04)	–	102.16(25.04)	1.83(0.4)	–	8.92(2.39)
	High	68.97(8.76)	–	175.37(17.1)	5.41(0.87)	–	14.56(1.83)
Place of ANCs	Government hospital	33.64(6.35)		100.96(12.9)	2.99(0.5)	–	9.69(1.27)
	Private hospital	98.3(13.41)	–	253.25(26.19)	7.45(1.58)	–	19.36(3.14)
Distance to ANC hospital	Less than 3kms	46.84(11.37)	–	139.31(31.67)	5.99(2.75)	–	15.31(4.27)
	3to 5kms	55.07(11.36)	–	143.17(24.21)	5.21(1.48)	–	14.15(3.21)
	6 and above	67.89(11.89)	–	172.67(21.75)	3.99(0.63)	–	11.55(1.61)
Who has taken decision on institutional delivery	Self/family planned	–	90.26(10.9)	137.01(14.16)	–	8.69(1.35)	12.93(1.95)
	Doctor advised/ Rushed to hospital due to EmOC	–	144.59(23.51)	178.36(24.01)	–	8.33(1.26)	13.13(1.93)
Type of delivery	Normal	–	76.76(11.45)	115.09(14.47)	–	6.32(1.09)	9.1(1.44)
	Caesarean/forceps	–	115.48(12.62)	183.44(19.77)	–	10.14(1.4)	15.76(2.1)
Type of hospital for delivery	Government hospital	–	41.76(6.02)	75.36(10.93)	–	4.76(1.02)	8.02(1.53)
	Government aided hospital	–	82.21(12.79)	109.6(14.53)	–	9.95(1.72)	14.46(2.77)
	Private hospital	–	230.07(23.13)	360.02(33.95)	–	14.44(2.45)	21.29(3.38)
Received JSY	Yes	–	59.67(10.42)	95.05(14.18)	–	7.53(1.62)	12.56(2.67)
	No	–	117.15(11.71)	182.3(17.69)	–	9(1.15)	13.22(1.61)

## Appendix 3

**Table 7:** Percentage of families with incidence of ‘catastrophic spending’ for maternal health care at different threshold by key predictors

*Variable*	*Categories*	Less than 15% level	15–25% level	More than 25% level
Age (in years)	Less than 25	71.08(5.01)	15.66(4.01)	13.25(3.74)
	25–29	78.43(4.09)	8.82(2.82)	12.75(3.32)
	30 and above	66.67(7.11)	17.78(5.76)	15.56(5.46)
Education level of women	Up to high school	73.15(4.28)	18.52(3.76)	8.33(2.67)
	Intermediate	83.33(6.92)	3.33(3.33)	13.33(6.31)
	Under graduation and above	70.65(4.77)	9.78(3.11)	19.57(4.16)
Religion	Hindu	74.35(3.17)	12.04(2.36)	13.61(2.49)
	Muslim	69.23(7.49)	17.95(6.23)	12.82(5.42)
Social group	SC/ST	72.50(7.15)	15.00(5.72)	12.50(5.30)
	OBC	71.43(4.96)	13.10(3.70)	15.48(3.97)
	General	75.47(4.20)	12.26(3.20)	12.26(3.20)
Per capita annual income	Below $390	33.33(10.54)	14.29(7.82)	52.38(11.17)
	$390.1 to $976	61.54(6.81)	19.23(5.52)	19.23(5.52)
	$976.1 and above	82.80(3.02)	10.83(2.49)	6.37(1.96)
Place of residence	Urban	73.53(3.8)	12.50(2.85)	13.97(2.98)
	Rural	73.40(4.58)	13.83(3.58)	12.77(3.46)
Social networks	Yes	82.61(8.08)	8.70(6.01)	8.70(6.01)
	No	72.46(3.11)	13.53(2.38)	14.01(2.42)
Mass media exposure	No	70.00(7.34)	15.00(5.72)	15.00(5.72)
	Yes	74.21(3.18)	12.63(2.42)	13.16(2.46)
Number of previous pregnancies	0	67.12(5.54)	20.55(4.76)	12.33(3.87)
	1	74.24(5.42)	10.61(3.82)	15.15(4.45)
	2 and above	78.02(4.36)	8.79(2.98)	13.19(3.57)
Last pregnancy registered with ANM	Yes	73.86(4.71)	12.50(3.55)	13.64(3.68)
	No	73.24(3.73)	13.38(2.87)	13.38(2.87)
Number of ANC visits	Less than 3	79.07(6.28)	9.30(4.48)	11.63(4.95)
	3 to 8	72.26(3.61)	13.55(2.76)	14.19(2.81)
	9 and above	71.88(8.08)	15.63(6.52)	12.50(5.94)
Quality of ANCs	Low	72.97(7.40)	10.81(5.18)	16.22(6.14)
	Medium	77.42(7.63)	16.13(6.72)	6.45(4.49)
	High	72.84(3.51)	12.96(2.65)	14.20(2.75)
Place of ANCs	Government hospital	77.48(3.41)	11.92(2.65)	10.60(2.51)
	Private hospital	65.82(5.37)	15.19(4.06)	18.99(4.44)
Distance to ANC hospital	Less than 3kms	68.57(7.96)	17.14(6.46)	14.29(6.00)
	3to 5kms	75.00(5.84)	12.50(4.46)	12.50(4.46)
	6 and above	73.33(4.34)	14.29(3.43)	12.38(3.23)
Who has taken decision on institutional delivery	Self/Family planned	76.15(3.75)	12.31(2.89)	11.54(2.81)
	Doctor advised/ Rushed to hospital due to EmOC	70.00(4.61)	14.00(3.49)	16.00(3.68)
Type of delivery	Normal	84.04(3.80)	5.32(2.33)	10.64(3.20)
	Caesarean/forceps	66.18(4.07)	18.38(3.33)	15.44(3.11)
Type of hospital for delivery	Government hospital	85.71(3.32)	8.04(2.58)	6.25(2.30)
	Government aided hospital	68.85(5.98)	16.39(4.78)	14.75(4.58)
	Private hospital	54.39(6.66)	19.30(5.27)	26.32(5.88)
Received JSY	Yes	75.00(5.14)	12.50(3.92)	12.50(3.92)
	No	72.78(3.55)	13.29(2.71)	13.92(2.76)
Total		73.48(2.92)	13.04(2.23)	13.48(2.26)

## Abbreviation

ANCs: Antenatal care services
DLHS: District level household survey
JSY: *Janani suraksha yojana*
MMR: Maternal mortality ratios
NFHS: National family health survey
NSS: National sample survey
OOPE: Out-of-pocket expenditure
PNCs: Postnatal care services
TME: Total maternity expenditure
WHO: World Health Organization
